# Appearing competent or moral? The role of organizational goals in the evaluation of candidates

**DOI:** 10.3389/fpsyg.2022.923329

**Published:** 2022-09-12

**Authors:** Kyriaki Fousiani, Jan-Willem Van Prooijen, Bibiana Armenta

**Affiliations:** ^1^Department of Organizational Psychology, University of Groningen, Groningen, Netherlands; ^2^Department of Social Psychology, Free University of Amsterdam, Amsterdam, Netherlands

**Keywords:** competence, morality, relational vs. instrumental goals, recommendation for recruitment, Big Two theory

## Abstract

The Big Two theoretical framework suggests that two traits, namely morality and competence, govern social judgments of individuals and that morality shows a primacy effect over competence because it has more diagnostic value. In this study we tested the primacy effect of morality in the workplace by examining how instrumental or relational goals of organizations might influence the importance of morality or competence of candidates during the hiring process. We hypothesized that the primacy effect of morality might hold when organizational goals are relational, but it might get reversed when organizational goals are instrumental. Supporting our hypothesis, in a field study and two experiments (both preregistered) we found that people perceive moral candidates as more appropriate for recruitment when an organization prioritizes relational goals (Studies 1, 2, and 3). In contrast, people perceive competent candidates as more appropriate for recruitment when an organization prioritizes instrumental goals (Studies 1 and 2). Perceived appropriateness of a candidate, in turn, predicts a stronger intention to recruit a candidate (Studies 2 and 3). These results provide evidence for a reversal of the primacy effect of morality in a work setting, and illuminate the important role of organizational goals in social judgments.

## Introduction

The importance of recruiting the right workers to organizations has been widely acknowledged (Combs et al., 2006). As a result, over the last two decades, the importance of employee knowledge, capabilities, and skills has become increasingly apparent in the hiring process (Breaugh and Starke, 2000). Indeed, given the “internationalization” of businesses and companies (Alvarado-Vargas et al., 2020) and the “war for talent” (Michaels et al., 2001) that comes along with it, identifying and hiring the most competent and qualified applicants seems to be a panacea. This suggests that if candidates want to make a good impression and stand a chance to get hired, they should come across as competent. Nevertheless, research suggests that competence is not the most highly valued trait in impression formation processes (Brambilla et al., 2012). More specifically, people tend to attach more value to morality (as opposed to competence)-oriented traits, referring to traits suggesting targets to be concerned with other’s well-being and to act according to generic moral norms (e.g., integrity, trustworthiness, ethicality, and sincerity), which have a stronger diagnostic value about a target’s character, enable observers to predict the target’s future behaviors, and protect themselves from threatening outcomes (Leach et al., 2007; Brambilla et al., 2012, 2013).

According to the Big Two theory (Abele and Bruckmüller, 2011; Abele and Wojciszke, 2013), a theoretical framework embedded in the person perception literature (Abele, 2003; Wojciszke, 2005; Abele and Bruckmüller, 2011; Abele et al., 2016, 2021; Koch et al., 2021), competence (also known as agency) and morality (also known as communion) are the two core dimensions underlying person and group impressions (see Judd et al., 2005; Abele et al., 2008; Brambilla et al., 2012; Koch et al., 2021).^1^ *Competence* involves qualities such as capability, intelligence, ambition, skillfulness, and efficiency, whereas *morality*, involves traits such as integrity, honesty, ethicality, trustworthiness, fairness, and sincerity (Abele, 2003, 2016, 2021; Wojciszke, 2005; Abele and Wojciszke, 2007; Abele and Bruckmüller, 2011; Rusconi et al., 2017, 2020; Unkelbach et al., 2020). There is abundant research underscoring the superiority and prevalence of morality over competence in person perception and impression formation (Ybarra et al., 2001; Abele and Bruckmüller, 2011; Wojciszke et al., 2011; Abele et al., 2021). Yet, very little is known about the prevalence of one or the other trait in impression formation about job candidates (Wojciszke and Abele, 2008; Zhu et al., 2021).

In the current study, we investigate the role of competence vs. morality in the perceived appropriateness of a candidate for a job (i.e., the overall impression formation about a candidate). We argue that whether competence or morality is a more important predictor of a candidate’s perceived appropriateness for a job varies as a function of the goals of an organization (*cf.* Zhu et al., 2021). As such, the current research was designed to make a novel contribution by investigating how an organization’s goals (e.g., profit maximization vs. sense of belonging) moderate the link between dimensions of person perception (morality vs. competence) and perceived appropriateness. We further predicted that perceived appropriateness of a candidate would in turn, relate to stronger recommendation for recruitment of the candidate. We investigate these issues in a field study with recruiters as participants and in two preregistered experiments.

Our study contributes to the literature in the following ways: First, it aims to extend the Big Two theory in particular (Abele and Bruckmüller, 2011; Abele and Wojciszke, 2013) and the person perception literature in general (Abele and Wojciszke, 2007; Abele et al., 2016, 2021) by testing the role of competence and morality it in the organizational context, where the literature is scarce (Wojciszke and Abele, 2008; Zhu et al., 2021). Moreover, this study informs the HRM literature about the antecedents and underlying processes that drive recruitment decisions in organizations (Kristof, 1996; Kristof-Brown, 2000). Finally, this contribution has important practical implications as well, as our findings might be of interest to HR practitioners who need to be aware of the role that both impression formation and organizational goals exert in recruitment decisions.

### The primacy of morality over competence

When people judge others or form an overall impression of them, they are faced with abundant information comprising their traits (Reeder and Brewer, 1979; Peeters, 1983; Neuberg and Fiske, 1987; Skowronski and Carlston, 1987). Empirical research has systematically demonstrated that morality carries more weight over competence, as it seems to have stronger diagnostic and predictive value when trying to recognize other people’s intentions (Ybarra et al., 2001; Abele and Bruckmüller, 2011; Wojciszke et al., 2011; Abele et al., 2021; Brambilla et al., 2021; Koch et al., 2021). More specifically, because morality includes traits that are profitable to others (e.g., traits that could potentially influence other people’s well-being), it is deemed more important in impression formation than competence, which includes traits that are self-profitable and can predominantly benefit the trait possessors themselves (Peeters, 1983, 2001; Peeters and Czapinski, 1990).

In line with this theorizing, Brambilla et al. (2012) found that morality is a better predictor of impression formation than competence because it informs people about whether or not a target is a threat. In a similar vein, Brambilla et al. (2013) found that information about morality is more diagnostic of behavioral intentions, and that people have less desire to interact with immoral targets whom they consider as a threat. Accordingly, in an experimental group setting, Leach et al. (2007) showed that information about the morality of a group was a stronger predictor of group appraisals than information about the competence of a group. In line with these findings, van der Lee et al. (2016) found that people perceive individuals with inferior morality (vs. inferior competence) as more different from their group, and more threatening to their image, and are therefore more likely to reject them. Also, Rudert et al. (2017) found that observers are more likely to socially exclude a person with inferior morality rather than a person with inferior competence. The above literature suggests that when evaluating others, morality has a primacy effect over competence, and more strongly influences people’s judgment of others (Peeters, 1983, 2001; Peeters and Czapinski, 1990; Rusconi et al., 2017, 2020; Unkelbach et al., 2020).

In this contribution, we aim to investigate the effects of morality vs. competence in work setting and see which judgment dimension dominates recruiters’ hiring decisions. Based on the Big Two theory (Abele and Bruckmüller, 2011; Abele and Wojciszke, 2013) and the primacy effect of morality that it involves (Peeters, 1983, 2001; Peeters and Czapinski, 1990), one would expect that morality is a stronger predictor than competence of a positive global impression of a candidate; in other words, recruiters would perceive a candidate of superior morality (as compared to superior competence) as more appropriate for a job. Consistent with this theorizing, Clokie and Fourie (2016) showed that when judging the employability of graduate students, employers valued students’ “soft” skills (also referred to as “people” skills or “interpersonal” skills), such as teamwork, communication ethics, courtesy, and dependability (Hager et al., 2002; Mitchell et al., 2010) to a greater extent than their “hard” skills (i.e., measurable skills that one can find in the job description and can be learned on the job training) for the mere reason that soft skills facilitate organizational functioning through good communication.

Despite the above literature, which is based on the Big Two theory (Abele and Bruckmüller, 2011; Abele and Wojciszke, 2013), in real life we often encounter situations where organizations forward competence (as opposed to morality)-related skills merely because they have more instrumental goals and they pursue maximization of gains (see The War for Talent; Chambers et al., 1998). For such organizations, one may question the perceived superiority of morality over competence. Indeed, organizations nowadays face an increasingly competitive climate, and therefore their focus often lies on the achievement of tangible outcomes (e.g., money), success, achievement, and upward mobility (Spence and Helmreich, 1983; Fletcher et al., 2008). For this reason, companies are often obsessed with employee competences and performance-related skills and invest in identifying and hiring as many top performers as possible (Kulkarni et al., 2015). One would expect that in such organizations, competence is valued as a key element to goal fulfillment (e.g., most questions in job interviews are about candidates’ competence-related skills). Accordingly, we argue that morality is not always dominant in impression formation; instead, the primacy effect of morality might get attenuated or even reversed in a work context, depending on the organizational goals. Below, we argue that when relational goals of an organization (e.g., harmonious relationships and sense of belonging) are salient, a candidate’s morality would be more influential in the hiring decision. In contrast, when instrumental goals of an organization (e.g., maximization of material and tangible gains) are salient, competence of a candidate would determine the hiring decision.

### Instrumental vs. relational goals

Although the Big Two theory assumes a primacy effect of morality over competence (Abele and Bruckmüller, 2011; see also, Brambilla et al., 2012, 2013, 2021), there are conditions under which perceived competence is more valued or informative than perceived morality (Hunt and Madhyastha, 2012). We claim that the extent to which competence or morality matter in person perception and evaluation largely depends on the goals and needs of the perceiver. For instance, people can be concerned with material and tangible outcomes, also known as instrumental goals (Luce and Raiffa, 1957; Thibaut and Kelley, 1959; Olson, 1965) or with relational (or symbolic) needs or goals, such as establishing positive social relationships, belonging to groups or entities, and the fulfillment of their psychological well-being (Tajfel and Turner, 1979; Turner et al., 1987; Deci and Ryan, 2000). While most people endorse both goals to some extent, they are conceptually distinct by focusing on different types of outcomes that are either material (instrumental) or symbolic (relational) in nature.

Organizations may have similar needs or goals. Instrumental organizational goals refer to objective factors such as pay, benefits, opportunity for advancement, maximization of profit and minimization of costs (see Lievens and Highhouse, 2003; see also Katz, 1960). Relational organizational goals refer to subjective and intangible factors such as relational harmony, need for social belonging, and person-organization relationships (see Bies and Moag, 1986; Tyler and Bies, 1990; King, 2009; Brienza and Bobocel, 2017). Although organizations – like individuals – usually strive for both types of goals to some extent, one type of goals might prevail over the other for various reasons, such as being more relevant for the survival and success of the organization. For instance, a real estate agency or an investment company, which are by definition attached to the attainment of specific, measurable, and tangible outcomes, might rely predominantly on the achievement of instrumental goals for their success (e.g., sell as many products as possible with the aim to maximize financial profit). Relational goals might be secondary in such organizations, as they are relevant only insofar they contribute to attaining these instrumental goals (e.g., a buyer might appreciate a pleasant interaction with the seller, but what matters for the organization’s survival is the products that they buy). In contrast, a kindergarten or a school, which have a strong social character (e.g., they provide services that involve social interactions and identity concerns), would need to prioritize the attainment of relationship-oriented goals for their success (e.g., invest in children well-being and sense of belonging). Although instrumental goals would still be important for the survival of such organizations (e.g., also school teachers require a salary), such organizations might focus more on the attainment of relational goals to measure their success.

In sum, instrumental and relational goals both matter in most organizations, but organizations may differ in the extent to which they prioritize one over the other (Zhu et al., 2021). The distinction between these types of goals is therefore important to recruiters, as organizational goals determine the type of skills and qualifications that are required from job candidates. For instance, job advertisements might accentuate the importance of hard skills (e.g., innovation skills or other mental and/or physical capabilities; see Karpinska et al., 2013, p: 1328) or soft skills (i.e., person rather than task-oriented skills and interpersonal rather than technical skills; see Krings et al., 2011 and Laker and Powell, 2011), depending on the organization’s goals. The types of goals that (implicitly or explicitly) are most central in the overall culture or identity of an organization may determine the primacy of competence or morality in impression formation about a candidate.

### The moderating role of instrumental vs. relational goals in the primacy of morality over competence

Despite the evidence on the primacy effect of morality over competence as, suggested by the Big-Two theory (Abele and Bruckmüller, 2011; see also Brambilla et al., 2021), in certain contexts, such as at work, the prevalence of one or the other dimension is debatable. Indeed, leading researchers of the Big Two theory have shown that the primacy effect of morality reverses under certain conditions. For instance, Wojciszke and Abele (2008) found that when an assessee’s competence is perceived as potentially profitable for the assessor (i.e., observer), an assessee’s competence becomes more important than their morality. Apparently, competence may outweigh morality in person perception when it serves perceivers’ goals, and this might be particularly the case in a work environment where the main goals are instrumental in nature.

In this study we suggest that competence, which according to the Big Two theory pertains to “getting ahead,” upward mobility, and success (Abele, 2003; Wojciszke, 2005; Abele and Wojciszke, 2007; Abele and Bruckmüller, 2011; Abele et al., 2016) might be more fitting in organizations that are concerned with achieving material and tangible outcomes. In contrast, morality which involves qualities that enable one to “get along,” and to come across as a credible person that others can trust and rely on might be more fitting in organizations that prioritize goals that involve relatedness, sense of belonging and social interactions. We therefore stated the following hypothesis:

*Hypothesis* 1: A candidate with superior competence (rather than superior morality) will be perceived as more appropriate (fitting) when the hiring organization has instrumental as opposed to relational goals. In contrast, a candidate with superior morality (rather than superior competence) will be perceived as more appropriate when the hiring organization has relational as opposed to instrumental goals.

Prior research has shown that perceiving a candidate as compatible with an organization in terms of values and traits reveals strong person–organization fit perceptions (P-O fit; Kristof, 1996; Kristof-Brown, 2000). Importantly, P–O fit perceptions seem to play a determinant role in recruiters’ decision-making processes in general (Gilmore and Ferris, 1989; Rynes and Gerhart, 1990) and in recruiters’ hiring decisions in particular (Cable and Judge, 1997; Kristof-Brown, 2000). More specifically, Cable and Judge (1997) and Kristof-Brown (2000) showed that recruiters’ P–O fit perceptions are the strongest predictor of recruitment recommendations. Interestingly, in a field study Higgins and Judge (2004) found that recruiters’ P-O fit perceptions mediated the effect of applicant’s influence tactics on recruitment recommendations. Based on the above, we stated the following hypothesis:

*Hypothesis* 2: Perceived appropriateness of a candidate will predict stronger hiring recommendations and will mediate the morality/competence by type of goals effect on recommendation for recruitment.

**Figure 1:**
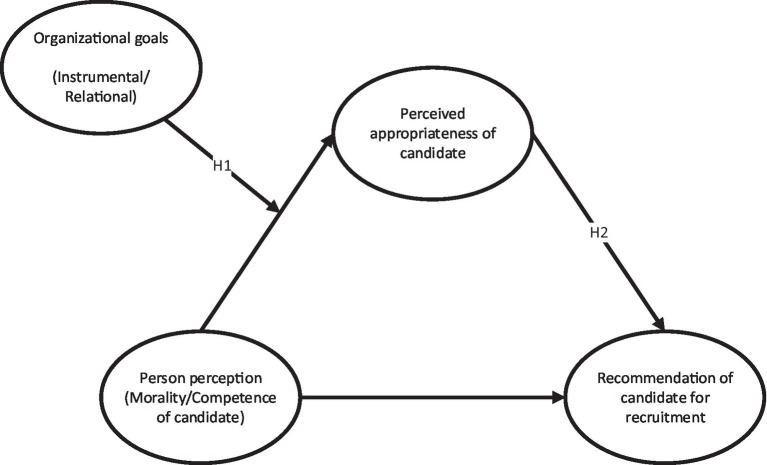
Hypothesized model.

A graphical illustration of the research model presented in Figure 1.

### Research overview

We conducted three studies to test the aforementioned hypotheses. Study 1 was a field study with recruiters and HR managers as participants. This study tested Hypothesis 1 by measuring the type of organizational goals (instrumental and relational) as perceived by the participants, and perception of competent and moral candidates as appropriate for recruitment. Studies 2 and 3 (both preregistered) tested both Hypothesis 1 and Hypothesis 2 (moderated mediation hypothesis). Study 2 was an experiment where we manipulated the morality vs. competence of a candidate and instrumental vs. relational goals of an organization in vignettes. To enable a straightforward test in a relatively simple design, in Study 2 we only included high morality/low competence vs. low morality/high competence conditions. Study 3 was designed to replicate the Study 2 findings using a full design, that manipulated morality and competence separately. In Study 1 we used M-Plus 8 (Muthén and Muthén, 2017) to perform confirmatory factor analysis (CFA) and to run a path analysis with latent variables. In Studies 2 and 3 we used SPSS 27 to run regression analyses.

## Study 1

### Method

#### Participants

A total of 260 participants (156 females; *M_age_* = 38.71, *SD* = 9.39) took part in an online study *via* Prolific. Of those, 181 participants were British, 13 were Irish, 11 were American, and 48 had another nationality. In total, 7 participants did not indicate their nationality. Moreover, all the participants were working as recruiters or HR managers or had recruiting experience/tasks. An *a-priori* power analysis revealed that 165 participants were required to achieve 95% power to detect a medium effect size (*f* = 0.25). Participants were paid £1.00.

#### Procedure

Participants first filled in an instrumental and relational organizational goals scale. The assessment of perceived appropriateness of candidate followed. Pre-selection of candidates is usually based on certain competence-related aspects (e.g., recruiters rely on candidates’ qualifications as presented in their CVs). Accordingly, while filling out these questionnaires, we instructed participants to bring to mind situations of job candidates who they believe that meet the job requirements as described in the respective job vacancies. Participants were debriefed and thanked upon completion of the study.

#### Measures

##### Instrumental goals

We developed an instrumental goals scale based on the Instrumental Concern Scale of Wilson and Putnam (1990). The original scale includes items that refer to the field of interpersonal negotiations in particular (e.g., “I want to get a better deal than my counterpart”), which we adapted to the specifics of this study (e.g., “the department/company where I work is concerned with how to make “good” deals). Not all the items of the original scale were possible to be adapted and therefore, not all the items were included in the adapted scale. Respondents were instructed to indicate the extent to which the department or company where they were working had instrumental goals or concerns. The scale consisted of 11 items (1 = *strongly disagree* to 7 = *strongly agree*; α = 0.92).

##### Relational goals

We developed a ten-item relational goals scale based on the Face Concerns Scale of Ting-Toomey and Oetzel (2001). Similar to the instrumental goals scale, the original scale includes items that refer to the negotiation context in particular (e.g., “Maintaining peace in my interaction with the other party was important to me”), and we adapted them to the specifics of this study (e.g., “The department/company where I work is concerned with maintaining peace in the interaction between people [employees, clients etc.]”). Respondents indicated the extent to which the department or company where they were working had relational goals or concerns (1 = *strongly disagree* to 7 = *strongly agree*; α = 0.94).

##### Perceived appropriateness of a moral vs. competent candidate

We measured the extent to which participants deemed moral or competent candidates as more appropriate for their company/department *via* a bipolar scale designed to tap participants relative preference for a moral or competent candidate. More specifically, participants were asked to indicate the extent to which, in their company/department, they perceive a candidate who has strong morality vs. competence-related traits as more appropriate [e.g., “In the company/department where I work, I would see as more appropriate (fitting) a candidate who is: 1 moral, 7 = competent]. We used a 5-item bipolar scale where one pole was presenting morality-related traits (1 = *moral, sincere, righteous, honest, fair*) and the other pole was presenting competence-related traits (7 = *competent, skillful, intelligent, capable, efficient*). The selection of the traits was based on the Agency and Communion Scale of Abele and Wojciszke (2007).^2^

##### Control variables

We controlled for participants’ age (in years) and gender (1 = male, 2 = female) as both variables have been found to influence recruiters’ hiring decisions (McMullin, 2011).

### Results

Correlations between the study variables, means, and standard deviations are presented in Table 1.

**Table 1:** Pearson correlations coefficients between study variables, means, and standard deviations (Study 1).

	1	2	3	*M (SD)*
Instrumental Goals^a^	1	−0.03	0.23^***^	4.70 (1.64)
Relational Goals^a^		1	−0.19^**^	4.92 (1.26)
Appropriateness of Candidate^b^			1	5.09 (1.22)

a Instrumental and relational goals were rated on a 7-point (1 = strongly disagree to 7 = strongly agree) Likert scale.

b Perceived appropriateness of candidate was rated on a 7-point (1 = moral candidate, 7 = competent candidate) bipolar scale.

** *p* < 0.01;

*** *p* < 0.001.

#### Preliminary analyses

To test whether instrumental and relational goals were distinct constructs we first conducted a Principal Component Analysis with Varimax rotation using SPSS 27. Two factors were extracted (relational goals and instrumental goals; Eigenvalues >1) which explained 32.80, and 31.07% of the variance, respectively. Most items had high loadings (|*f*_ij_| > 0.50) on the predicted factors except of two items of the instrumental goals scale which had lower loadings. Moreover, one item of the instrumental scale loaded on the relational goals scale. Accordingly, these three items were excluded from further analysis. The reliability of the instrumental goals scale, after excluding these items was.96. The factor loading matrix is presented in Appendix A.

We then conducted a CFA to ensure that our variables were distinct from one another. In the analysis, we included instrumental and relational goals (excluding the two instrumental and the one relational goal items that did not load as expected in the factor analysis) and perceived appropriateness of candidate. The model had good fit (*χ^2^* = 421.83, *df* = 227, *p* < 0.001; RMSEA = 0.06 [CI_90_ = 0.05; 0.07]; CFI = 0.96; SRMR = 0.06).

#### Hypothesis testing

Age and gender of participants served as control variables^3^. We tested the effect of organizational goals on perceived appropriateness of moral versus competent candidates *via* a path analysis with latent variables, using M-Plus 8 (Muthén and Muthén, 2017). Relational and instrumental goals were the predictors while appropriateness of moral versus competent candidates was the dependent variable. We found the effect of relational goals on the perceived appropriateness of a moral vs. competent candidate to be significant and negative. Given that ratings of morality/competence were on a 7-point scale where 1 = moral candidate and 7 = competent candidate, this effect suggests that participants perceived a moral candidate as relatively more appropriate to the extent that the organization’s goals were more strongly relational. Moreover, the effect of instrumental goals on the perceived appropriateness of a moral vs. competent candidate was significant and positive, suggesting that participants perceived a competent candidate as more appropriate to the extent that the organization’s goals were more strongly instrumental (see Table 2 for the relevant statistics). These results show that participants working in a company with stronger relational goals perceived moral candidates as more appropriate whereas participants working in a company with stronger instrumental goals perceived competent candidates as more appropriate. These findings provide support for Hypothesis 1.

**Table 2:** Results on perceived appropriateness of a moral or competent candidate using latent variables (Study 1).

Predictor	*B*	*SE*	95% *CI*
Relational Goals	−0.26^***^	0.08	−0.39; −0.14
Instrumental Goals	0.21^**^	0.07	0.09; 0.32
Age	−0.002	0.008	−0.02; 0.01
Gender	0.03	0.16	−0.22; 0.29

Relational and instrumental goals were rated on a 7-point (1 = strongly disagree to 7 = strongly agree) Likert scale. Perceived appropriateness of moral vs. competent candidate was rated on a 7-point (1 = moral candidate, 7 = competent candidate) bipolar scale.

** *p  *< 0.01;

*** *p  *< 0.001.

### Discussion

Study 1 was a field study aiming to test recruiters’ perception of moral vs. competent candidates as more appropriate depending on the organizational goals (instrumental and relational) of the company where they work. All participants were either recruiters, HR managers, or had managerial positions with hiring responsibilities. The results showed that the more strongly organizational goals are considered to be relational, the more a moral candidate is perceived to be appropriate for hiring. Moreover, the more organizational goals are considered to be instrumental, the more a competent candidate is perceived to be appropriate for hiring. These results provided support for Hypothesis 1.

Study 1, however, includes two major limitations: First, it was a cross-sectional study with all the data being collected in the same wave. This makes the results vulnerable to potential common method bias (Podsakoff et al., 2012). The cross-sectional research design of Study 1 also was the main reason for not testing Hypothesis 2 [i.e., the mediating role of perceived appropriateness in the relationship between candidate traits (competence and morality) by organizational goals and recommendation for recruitment]. Second, Study 1 measured the perceived appropriateness of moral vs. competent candidates in one single scale that combined the assessment of both constructs. More specifically, participants were asked to rate the appropriateness of a moral vs. competent candidate on a bipolar scale where morality-related characteristics were on the one pole of the scale and competence-related characteristics on the other. Study 2 was a follow-up study aiming to replicate the findings of Study 1 in an experimental design, and also test Hypothesis 2. Importantly, in an attempt to address some of the limitations of Study 1, Study 2 manipulated candidate’s person perception (morality vs. competence) and assessed perceived appropriateness of candidate on a separate measure.

## Study 2

### Methods

#### Participants

A total of 318 participants^4^ (193 females; *M_age_* = 27.58, *SD* = 11.83) living in the Netherlands took part in this study. Of those, 147 participants were Dutch, 43 were German, 16 were British, and 112 had another nationality. Moreover, 84 participants had a full-time job, 120 participants had a part-time job, and 110 were unemployed.^5^ Of the participants, 106 had finished high-school, 128 had a bachelor’s degree and 80 had a Master’s or a PhD. In total, 4 participants did not indicate their occupational or educational status. An *a-priori* power analysis revealed that 269 participants were required to achieve 80% power to detect a medium effect size (*f* = 0.25). The study was preregistered in Open Science Framework: https://osf.io/gyzwx/?view_only=7da67f94b23746a09281fb45155d67f0.^6^

#### Experimental design and procedure

Graduate students recruited participants by using their student or work environment and their personal network. Potential participants were approached *via* e-mail, social media (e.g., Facebook, LinkedIn, Twitter) or face-to face contact and were requested to take part in an online study. Participants were not paid. The survey was programmed in Qualtrics.

We manipulated instrumental vs. relational goals of the organization in vignettes. Participants read a job vacancy and were asked to take the perspective of an HR manager, who was supposed to hire one of the candidates that had applied. More specifically, participants read that the company they are working at is seeking a candidate to fulfill the Management Consultant role. In the instrumental goals condition, the vacancy pointed out the need for fulfilling instrumental tasks, such as “designing a strategic plan for the maximization of the company’s revenues and profit, identifying financial problems and designing profitable strategies, and developing processes, routines, and tools to optimize the profit of the company.” In the relational goals condition, the vacancy pointed out the need for fulfilling relational tasks, such as “designing a strategic plan for the maximization of the employees’ well-being and sense of belonging to the company, identifying collaboration problems between company members, and designing conflict resolution strategies, and developing processes, routines, and tools to optimize relationship quality between the members of the company” (see Online Supplementary material for the complete vignettes).

Manipulation checks followed directly after the presentation of the vacancy descriptions. More specifically, we asked participants to indicate the extent to which the job vacancy had a focus on (a) A sense of belonging, fair solutions, communication (manipulation check item for relational goals); (b) Revenues and financial profit maximization [manipulation check item for instrumental goals” (1 = *not at all true*, 7 = *absolutely true*)].

For ecological validity, but also to make the manipulations more convincing, we informed participants that four candidates were shortlisted based on their qualifications. Among the four, two candidates were invited for an interview based on their CVs; therefore, the race would be decided among two candidates. Laustsen and Bor (2017) adopted a similar approach for the manipulation of morality and competence of political candidates. Thereafter, we presented participants with a brief description of each of the two candidates. Similar to Laustsen and Bor (2017), we only manipulated the competence/morality of one of the candidates while the other candidate was presented as having moderate competence and morality (see Online Supplementary material for the description of the candidate).

For the manipulation of competence/morality of the candidate we used adapted versions of the vignettes of Laustsen and Bor (2017): The candidate was presented as having superior competence but inferior morality (“*Mr de Vries has many years of experience in management. He has designed an excellent initiative to identify and analyze company’s interests and translate them into projects that assure their realization. His ideas have been widely praised for their insights, effectiveness, and originality. However, in his career so far, Mr de Vries has proved to be a controversial person. His colleagues describe him as not the most honest man who sometimes breaks his word. Mr de Vries is also accused of being, at times, disrespectful or impolite*”) vs. inferior competence but superior morality (“*Mr de Vries has mostly worked as a security engineer and has very little experience in management. He frequently recites an initiative to identify and analyze company’s interests and translate them into projects that assure their realization. However, his ideas have been widely judged as shallow, ineffective, and naïve. However, in his career so far, Mr de Vries has proved to be a reputable person. His colleagues describe him as an honest man who always keeps his word. Mr de Vries is also praised for treating others with respect and politeness*”). The order of presentation of competence or morality information was randomized in order to avoid order-effect biases (Perreault, 1975).

Manipulation checks followed directly after the morality/competence information. Participants were asked to rate the extent to which they perceived each candidate as competent, intelligent, skilled (competence manipulation check items; α = 0.82), moral, sincere, honest (morality manipulation check items; α = 0.95; 1 = *not at all*, 7 = *a lot*) similar with Laustsen and Bor (2017).

#### Measures

##### Perceived appropriateness of candidate (global impression of the candidate)

We developed a 3-item measure based on the P-O fit scale of Cable and Judge (1997) and the global impression scale of Brambilla et al. (2012): “To what extent do you think that Mr. de Vries fulfills the criteria for this job?”; “To what extent do you think that Mr. de Vries is an appropriate candidate for this position?”; “To what extent is your global impression of Mr. de Vries favorable?” (1 = *not at all*, 7 = *to a great extent*; α = 0.70).

##### Recommendation of the candidate for hire

We developed a measure based on the recommendation for hiring scale of Higgins and Judge (2004). We asked participants to indicate whether or not they would recommend the applicant for recruitment with two items: “Would you recommend Mr. de Vries for this position?”; “Would you offer this job to Mr. de Vries?” (1 = *absolutely not*, 7 = *absolutely yes;* α = 0.93). ^7^^,^^8^

##### Control variables

Similar to Study 1, we controlled for participants’ age and gender (1 = male, 2 = female).

### Results

#### Manipulation checks

We ran a Multivariate Analysis of Variance (MANOVA) with the type of organizational goals (instrumental vs. relational) as the independent variable and the instrumental and relational goals manipulation check items as the dependent variables. The multivariate effect of type of goals was significant *F*(2,315) = 295.70, *p* < 0.001, *η^2^* = 0.65 and showed that participants perceived the goals of the vacancy as more instrumental in the instrumental (*M* = 6.53, *SD* = 0.85) than relational (*M* = 3.25, *SD* = 1.68) conditions, *F*(1,316) = 488.34, *p* < 0.001, *η^2^* = 0.61. In contrast, participants perceived the goals as more relational in the relational (*M* = 5.64, *SD* = 1.49) as opposed to the instrumental (*M* = 2.56, *SD* = 1.51) condition, *F*(1,316) = 335.86, *p* < 0.001, *η^2^* = 0.52.

We then ran a simple MANOVA with the morality/competence manipulation as the independent variable, and the morality and competence manipulation check scales as the dependent variables. The multivariate effect of morality/competence manipulation was significant *F*(2,315) = 238.58, *p* < 0.001, *η^2^* = 0.60 and showed that participants perceived the candidate in the high morality/low competence condition as more moral (*M* = 5.54, *SD* = 1.10) as compared with the low morality/high competence condition (*M* = 2.93, *SD* = 1.33), *F*(1,316) = 365.34, *p* < 0.001, *η^2^* = 0.53. Moreover, participants perceived the candidate in the low morality/high competence condition as more competent (*M* = 5.40, *SD* = 1.06) as compared to the high morality/low competence condition (*M* = 4.31, *SD* = 1.03), *F*(1,316) = 86.03, *p* < 0.001, *η^2^* = 0.21. Finally, we ran a 2 × 2 MANOVA with goals and morality/competence as predictors and the morality and competence scales as outcome variables. The interaction effect did not come out significant *F*(2,315) = 0.95, *p* = 0.39, *η^2^* = 0.006. We conclude that the manipulations worked as intended.

#### Hypothesis testing

**Figure 2:**
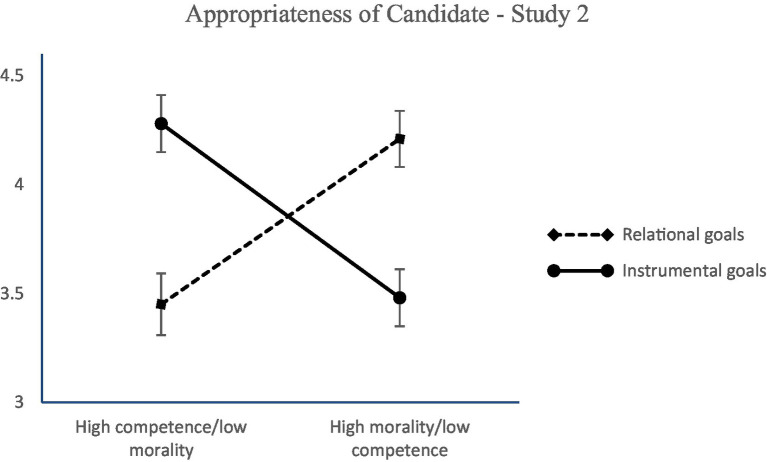
Perceived appropriateness of a candidate as a function of morality/competence and type of goals (Study 2). Ratings were on a 7-point scale (1 = *not at all*, 7 = *to a great extent*). Error bars represent standard errors.

We ran a moderated mediation analysis in Process (Hayes, 2013, 2018). We requested a 95% bias-corrected interval based on 5,000 bootstrap samples. Competence/morality (−1 = high competence but low morality, 1 = low competence but high morality) was the independent variable, type of goals (−1 = relational, 1 = instrumental) was the moderator, perceived appropriateness of the candidate was the mediator, and intention for recommendation of the candidate was the dependent variable. Age and gender were included as control variables. The overall model was significant *R*^2^ = 0.10, *F* (5,308) = 6.84, *p* < 0.001. The main effect of competence/morality on perceived appropriateness of the candidate was not found to be significant. Similarly, the main effect of type of goals did not prove to be significant. However, the interaction effect on perceived appropriateness of the candidate came out significant (see Table 3 for the relevant statistics) and showed that people perceive a candidate of high competence (rather than high morality) as more appropriate for hire when the goals of the job are instrumental (*b* = −0.40, *SE* = 0.09, *p* < 0.001; 95% CI [−0.58; −0.21]). Moreover, participants perceived a candidate of high morality (rather than high competence) as more appropriate when the goals of the job are relational (*b* = 0.39, *SE* = 0.10, *p* = 0.001; 95% CI [0.20; 0.58]), Δ*R*^2^ = 0.10, *F* (1,308) = 33.94, *p* < 0.001 (see Figure 2). These results support Hypothesis 1.

**Table 3:** Regression analyses results on perceived appropriateness of a candidate and recommendation for recruitment (Study 2).

Predictor	*B*	*SE*	*t*	*p*	95% *CI*
Perceived Appropriateness of Candidate (Mediator)^d^					
Constant	3.90	0.30	12.88	<0.001	3.31; 4.50
Morality/Competence^a^	−0.004	0.07	−0.06	0.95	−0.14; 0.13
Type of Goals^b^	0.04	0.07	0.52	0.60	−0.10; 0.17
Morality/Competence^a^ × Type of Goals^b^	−0.40	0.07	−5.83	<0.001	−0.53; −0.26
Age	0.001	0.006	0.01	0.99	−0.03; 0.01
Gender^c^	−0.04	0.13	−0.28	0.78	−0.01; 0.01
Recommendation Intention (Dependent Variable)^e^					
Constant	−0.14	0.26	−0.55	0.58	−0.64; 0.36
Morality/Competence^a^	−0.02	0.05	−0.53	0.59	−0.11; 0.07
Type of Goals^b^	−0.04	0.05	−0.90	0.37	−0.13; 0.05
Appropriateness of Candidate^d^	0.99	0.04	25.69	<0.001	0.92; 1.07
Morality/Competence^a^ × Type of Goals^b^	−0.05	0.05	−1.08	0.28	−0.15; 0.04
Age	−0.001	0.004	−0.26	0.79	−0.01; 0.01
Gender^c^	−0.02	0.09	−0.23	0.82	−0.20; 0.16
Conditional Indirect Effects					
Mediator	Goals	*B*	Boot*SE*	Boot 95% *CI*	
Appropriateness of Candidate^d^	Relational	0.39	0.10	0.19	0.59
	Instrumental	−0.40	0.09	−0.57	−0.22

a Morality/Competence was codes as: −1 = high competence but low morality, 1 = low competence but high morality.

b Type of goals was coded as: −1 = relational, 1 = instrumental.

c Gender: 1 = male, 2 = female.

d Perceived appropriateness of candidate was rated on a 7-point (1 = not at all, 7 = to a great extent) Likert scale.

e Intention for recommendation for recruitment was rated on a 7-point (1 = absolutely not, 7 = absolutely yes) Likert scale.

Perceived appropriateness of the candidate (*M* = 3.86, *SD* = 1.29) was positively related to the intention for recommendation of the candidate (*M* = 3.65, *SD* = 1.49; *r* = 0.78, *p* < 0.001). Moreover, although the direct effect of morality/competence on recommendation for recruitment was not significant, the indirect effect was significant resulting in a full mediation (see Table 3 for the relevant statistics). The overall moderated mediation model was supported with the index of moderated mediation = −0.78, *SE* = 0.13, 95% CI [−1.05; −0.52]. These results corroborate Hypothesis 2 and support the idea that perceived appropriateness mediates the link between morality/competence, organizational goals and recruitment intentions.

### Discussion

Study 2 experimentally tested the moderated effect of type of goals of an organization on the relationship between a candidate’s morality or competence and recommendation for recruitment through perceived appropriateness (fitness). Replicating and extending Study 1, the findings supported Hypothesis 1 and showed that the primacy effect of morality might be reversed when organizational goals are instrumental. Further, this study provided full support for moderated mediation (Hypothesis 2), such that the interaction effect between candidate’s traits (competence/morality) and organizational goals on perceived appropriateness of a candidate subsequently predicts recommendation for recruitment. A limitation of Study 2, however, is that the experimental design only compared candidates who scored high in one but low in the other dimension (e.g., high morality but low competence vs. high competence but low morality). This is consistent with prior research that has conceptualized morality (as a subdimension of warmth) and competence as antagonistic poles of a single dimension (Bakan, 1966). Recent literature, however, conceives morality and competence as two separate dimensions which are (nearly) orthogonal, meaning that a high rating in one dimension can be companied with either a low or high rating in the other dimension (Fiske et al., 2002). Yet, there is evidence that the two dimensions are not orthogonal (Abele and Wojciszke, 2014) and can relate positively (see “halo” and “horn” effects; Dion et al., 1972) or negatively to each other (see compensation effect; Wojciszke, 1994; Judd et al., 2005; Kervyn et al., 2012). Some studies even find a curvilinear relationship (Imhoff and Koch, 2017). To achieve a better understanding of the role of morality and competence in hiring decisions when instrumental or relational goals are prevalent, we conducted an experiment where we manipulated morality (high vs. low) and competence (high vs. low) as separate dimensions. Hence, Study 3, does not only test the two-way interaction between morality/competence (as one dimension) and type of goals, but also tests the three-way interaction between type of goals, morality, and competence. The purpose of the three-way interaction is exploratory and goes beyond the scope of the current contribution, and therefore we did not state specific hypotheses about it.

Finally, an additional limitation of Study 2 is the heterogeneity of the sample in terms of the participants’ employment status. Indeed, a large number of participants were full-time employees, an even larger number were part-time employees, while one third of the participants were unemployed. We consider this as a limitation of the study, as people’s employment status might influence the extent to which they identify with the role they are assigned while reading the study vignettes, and it might determine the judgments they make about job candidates. To address this limitation, Study 3 recruited predominantly full-time employees.

## Study 3

### Methods

#### Participants

A total of 400 British participants, living in the United Kingdom, took part in this study. Of the participants, 6 were removed because they had either indicated that they did not complete the study truthfully or they had not fully completed the study. Of those (207 females, *M_age_* = 38.73, *SD* = 8.63), 376 had a full-time job, 13 participants had a part-time job, and 5 were unemployed. Moreover, 125 had finished high-school, 171 had a bachelor’s degree and 97 had a Master’s or a PhD. In total, seven participants did not indicate their occupational or educational status. An *a-priori* power analysis revealed that 400 participants were required to achieve 95% power to detect a medium effect size (*f* = 0.25). This study was preregistered on OSF^9^

#### Experimental design and procedure

Participants were recruited *via* Prolific and were paid £0.90. We manipulated instrumental vs. relational goals using the same vignettes as in Study 2. Moreover, we used the same vignettes as Study 2 to manipulate morality and competence, with the difference that we included additional conditions to achieve a complete design: morality (high vs. low) and competence (high vs. low). Therefore, we had a 2 × 2 × 2 between-participants experimental design. The two candidates were given typical British names. Manipulation checks followed and were identical to those of Study 2 [competence manipulation check scale; α = 0.91; (morality manipulation check scale; α = 0.97)].

#### Measures

We used the same perceived appropriateness of the candidate and recommendation of the candidate scales (α = 0.95 and α = 0.98 respectively) as in Study 2 after adding one item to each scale (see Online Supplementary material). Similar to Studies 1 and 2, we controlled for participants’ age and gender.

### Results

#### Manipulation checks

The multivariate effect of type of goals was significant *F*(2,391) = 722.85, *p* < 0.001, *η^2^* = 0.79 and showed that participants perceived the goals of the vacancy as more instrumental in the instrumental (*M* = 6.76, *SD* = 0.76) than relational (*M* = 2.71, *SD* = 1.55) conditions, *F*(1,392) = 1077.39, *p* < 0.001, *η^2^* = 0.73. In contrast, participants perceived the goals as more relational in the relational (*M* = 6.15, *SD* = 1.05) as opposed to the instrumental (*M* = 2.25, *SD* = 1.43) condition, *F*(1,392) = 960.02, *p* < 0.001, *η^2^* = 0.71.

We then ran a 2 (morality: high vs. low) × 2 (competence: high vs. low) multivariate ANOVA with the manipulation check scales for morality and competence as dependent variables. The multivariate effect of morality was significant *F*(2,389) = 463.85, *p* < 0.001, *η^2^* = 0.71 and showed that participants perceived the candidate as more moral in the high morality condition (*M* = 5.90, *SD* = 0.92) than in the low morality condition (*M* = 2.55, *SD* = 1.23), *F*(1,390) = 928.40, *p* < 0.001, *η^2^* = 0.70. However, the effect of morality on perceived competence of the candidate was also significant *F*(1,390) = 79.92, *p* < 0.001, *η^2^* = 0.17 and showed that participants in the high morality condition (*M* = 5.38, *SD* = 1.07) were perceived as more competent than those in the low morality condition (*M* = 4.35, *SD* = 1.46).

The multivariate effect of competence also came out significant *F*(2,389) = 172.62, *p* < 0.001, *η^2^* = 0.47. As expected, participants perceived the candidate as more competent in the high competence (*M* = 5.81, *SD* = 0.89) as compared to the low competence condition (*M* = 4.00, *SD* = 1.16), *F*(1,390) = 329.99, *p* < 0.001, *η^2^* = 0.46. Unexpectedly, the effect of competence on perceived morality of the candidate was also significant *F*(1,390) = 5.16, *p* = 0.02, *η^2^* = 0.01 and showed that a candidate in the high competence condition (*M* = 4.53, *SD* = 1.90) was perceived as more moral than a candidate in the low competence condition (*M* = 3.96, *SD* = 2.05). Finally, the morality × competence multivariate interaction effect came out significant *F*(2,389) = 5.36, *p* = 0.005, *η^2^* = 0.03 and showed that participants perceived a candidate in the high competence and high morality condition as more competent (*M* = 6.05, *SD* = 0.69) than a candidate in the high competence and low morality condition (*M* = 5.51, *SD* = 1.01), *F*(1,390) = 10.74, *p* = 0.001, *η^2^* = 0.03. The interaction effect on perceived morality of a candidate was not significant *F*(1,390) = 1.13, *p* = 0.29, *η^2^* = 0.003. Although moral candidates were also perceived as more competent, and vice versa (which is in line with the halo-effect; Nisbett and Wilson, 1977), the expected main effects had much stronger effect sizes than the unintended effects, and therefore we regard the morality and competence manipulations as satisfactory for the present purposes.

We then ran a 2 × 2 × 2 MANOVA with morality, competence and goals as predictors and the morality and competence scales as outcome variables. None of the interaction effects proved to be significant *F*s < 1. We conclude that the manipulations worked as intended.

#### Hypothesis testing

We ran a 2 × 2 × 2 moderated mediation analysis in Process (Hayes, 2013, 2018). In the analysis, type of goals (−1 = relational, 1 = instrumental), morality (−1 = low morality, 1 = high morality), and competence (−1 = low competence, 1 = high competence) were the predicting variables, appropriateness of candidate was the mediator and recommendation for recruitment was the dependent variable. Age and gender of participants were added as control variables. The overall model was significant *R*^2^ = 0.70, *F* (9,384) = 97.85, *p* < 0.001. The main effect of type of goals did not prove to be significant. The main effect of morality on perceived appropriateness of the candidate was significant and showed that participants perceived a highly moral person as more appropriate for a job. Similarly, the main effect of competence was significant and showed that participants perceived a highly competent candidate as more appropriate for hire.

**Figure 3:**
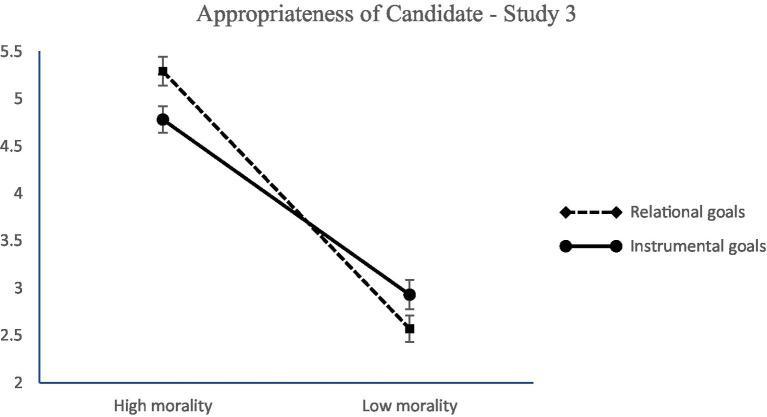
Perceived appropriateness of a candidate as a function of morality and type of goals (Study 3). Ratings were on a 7-point scale (1 = *not at all*, 7 = *to a great extent*). Error bars represent standard errors.

**Figure 4:**
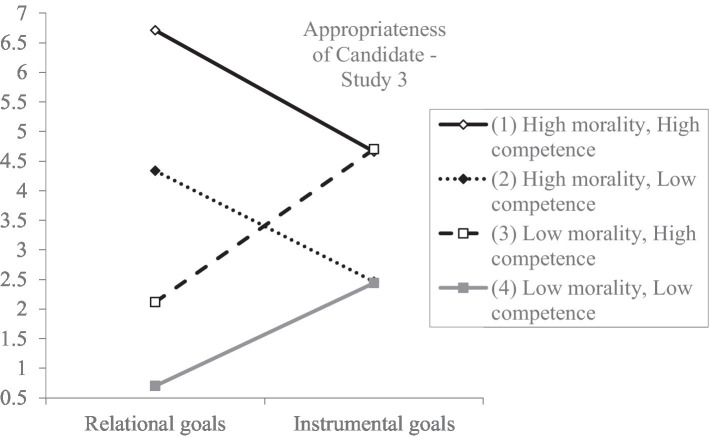
Perceived appropriateness of a candidate as a function of type of goals, morality, and competence (Study 3). Ratings were on a 7-point scale (1 = *not at all*, 7 = *to a great extent*). Error bars represent standard errors.

Importantly, the morality × goals interaction effect on perceived appropriateness of the candidate came out significant (see Table 4 for the relevant statistics) and showed that people perceive a candidate of high morality as more appropriate when the goals of the job are relational (*b* = −0.19, *SE* = 0.07, *p* < 0.01; 95% CI [−0.34; −0.05]) than when goals are instrumental (*b* = 0.30, *SE* = 0.07, *p* < 0.001; 95% CI [0.15; 0.45]) Δ*R*^2^ = 0.02, *F* (1,385) = 21.40, *p* < 0.001 (see Figure 3). Unexpectedly, the competence × goals interaction effect on perceived appropriateness was not significant (see Table 4 for the relevant statistics). These results partly support Hypothesis 2. Furthermore, the morality × competence interaction came out significant and showed that highly competent candidates are deemed as more appropriate for hire when they are also high in morality (*b* = 1.14, *SE* = 0.08, *p* < 0.001; 95% CI [0.99; 1.29]) rather than low in morality (*b* = 0.92, *SE* = 0.07, *p* < 0.001; 95% CI [0.77; 1.07]) Δ*R*^2^ = 0.004, *F* (1,385) = 4.47, *p* < 0.05. Finally, the morality × competence × type of goals interaction was significant and showed that participants perceived a candidate who is high in competence and low in morality to be more appropriate for a job when organizational goals are instrumental (*b* = 0.50, *SE* = 0.12, *p* < 0.001; 95% CI [0.27; 0.72]), and a candidate who is high both in morality and in competence when organizational goals are relational (*b* = −0.23, *SE* = 0.10, *p* = 0.02; 95% CI [−0.43;-0.03]) Δ*R*^2^ = 0.005, *F* (1,385) = 5.71, *p* = 0.02 (Figure 4).

**Table 4:** Regression analysis results on the effect of morality and competence on perceived appropriateness of a candidate and recommendation for recruitment as a function of type of goals (Study 3).

Predictor	*B*	*SE*	*t*	*p*		95% *CI*	
Perceived Appropriateness of Candidate (Mediator)^e^							
Constant	3.77	0.29	13.01	<0.001		3.20; 4.34	
Type of Goals^a^	0.05	0.05	0.84	0.40		−0.06; 0.15	
Morality^b^	1.02	0.05	19.23	<0.001		0.92; 1.12	
Competence^c^	1.03	0.05	19.21	<0.001		0.93; 1.13	
Morality^b^ × Type of Goals^a^	−0.23	0.05	−4.37	<0.001		−0.34; −0.13	
Competence^c^ × Type of Goals^a^	0.08	0.05	1.56	0.12		−0.02; 0.19	
Morality^b^ × Competence^c^	0.11	0.05	2.11	0.03		0.01; 0.22	
Morality^b^ × Competence^c^ × Type of Goals^a^	−0.13	0.05	−2.39	0.02		−0.23; −0.02	
Age	−0.004	0.006	−0.56	0.57		−0.02; 0.01	
Gender^d^	0.17	0.10	1.67	0.10		−0.03; 0.38	
Recommendation Intention (Dependent Variable)^f^							
Constant	−0.41	0.24	−1.70	0.09		−0.89; 0.06	
Type of Goals^a^	0.02	0.04	0.43	0.67		−0.13; 0.09	
Morality^b^	0.05	0.05	1.01	0.31		−0.05; 0.15	
Competence^c^	−0.02	0.05	−0.42	0.50		0.14; 0.07	
Appropriateness of Candidate^e^	1.06	0.04	29.81	<0.001		0.99; 1.13	
Morality^b^ × Type of Goals^a^	0.01	0.04	0.30	0.77		−0.06; 0.08	
Competence^c^ × Type of Goals^a^	0.09	0.04	2.37	0.02		0.01; 0.16	
Morality^b^ × Competence^c^	0.14	0.04	3.78	0.002		0.07; 0.21	
Morality^b^ × Competence^c^ × Type of Goals^a^	0.01	0.04	−0.001	0.99		−0.07; 0.07	
Age	−0.005	0.004	−1.15	0.25		−0.01; 0.004	
Gender^d^	0.08	0.07	1.14	0.25		−0.06; 0.21	
Conditional Indirect Effects for Morality							
Mediator	Goals	Competence	Morality	*B*	Boot*SE*	Boot 95% *CI*	
Appropriateness of Candidate^e^	Relational	High	High	−0.25	0.08	−0.40	−0.09
	Instrumental	High	Low	0.53	0.14	0.24	0.80
	Relational	Low	High	−0.05	0.12	−0.40	0.09
	Instrumental	Low	Low	0.08	0.10	−0.11	0.27

a Type of goals was coded as: −1 = relational, 1 = instrumental.

b Morality was coded as: −1 = low morality, 1 = high morality.

c Competence was coded as: −1 = low competence, 1 = high competence.

d Gender: 1 = female, 2 = male.

e Perceived appropriateness of candidate was rated on a 7-point (1 = not at all, 7 = to a great extent) Likert scale.

f Intention for recommendation for recruitment was rated on a 7-point (1 = absolutely not, 7 = absolutely yes) Likert scale.

As in Study 2, perceived appropriateness of the candidate (*M* = 3.88, *SD* = 1.86) was positively related with intention for recommendation of the candidate (*M* = 3.69, *SD* = 2.13; *r* = 0.94, *p* < 0.001). Furthermore, perceived appropriateness of the candidate had a significant and positive effect on the intention to recommend the candidate for recruitment. The above results partly support Hypothesis 2. Neither the main effect of morality, competence or type of goals on recommendation for recruitment were significant.

**Figure 5:**
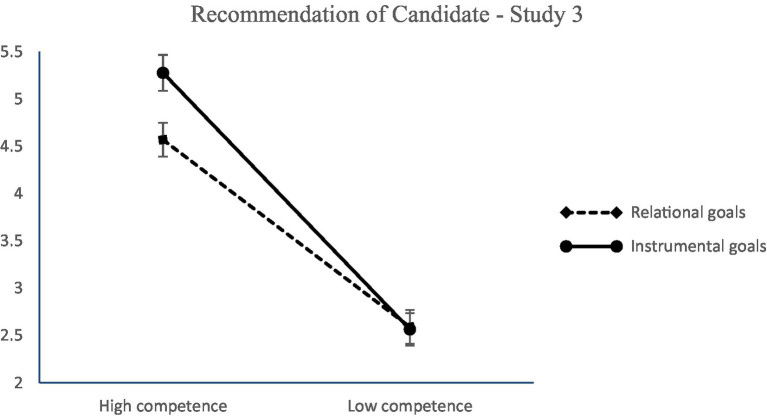
Intention to recommend a candidate as a function of competence and type of goals (Study 3). Ratings were on a 7-point scale (1 = *not at all*, 7 = *to a great extent*). Error bars represent standard errors.

Although beyond our hypotheses, it is worth reporting that the competence × type of goals interaction on recommendation for hire was significant. Results showed that people have a stronger intention to recommend for hire a candidate of high competence when the goals of the job are instrumental (*b* = 0.11, *SE* = 0.05, *p* < 0.05; 95% CI [0.001; 0.21]) than when goals are relational (*b* = −0.06, *SE* = 0.05, *p* > 0.05; 95% CI [0.16; 0.04]), Δ*R*^2^ = 0.002, *F* (1,384) = 5.37, *p =* 0.02 (see Figure 5). Moreover, the morality × competence interaction on recommendation for recruitment was also significant and showed that participants recommend for recruitment to lower extent candidates who are low both in competence and morality than candidates who are high in one or both dimensions (*b* = −1.16, *SE* = 0.06, *p* < 0.01; 95% CI [−0.28; −0.40]) Δ*R*^2^ = 0.004, *F* (1,384) = 14.37, *p <* 0.001. Finally, and most importantly, morality × competence × type of goals had an indirect effect on recommendation for recruitment through perceived appropriateness of candidate (full mediation; see Table 4 for the relevant statistics). The overall moderated mediation model was supported with the index of moderated mediation (IMM) = −0.13, *SE* = 0.06, 95% CI [−0.25; −0.02].

### Discussion

Study 3 was a follow-up study aiming to replicate the results of Study 2 while manipulating morality and competence separately. Accordingly, besides the competence × type of goals and morality × type of goals interactions, Study 3 further tested the three-way interaction between type of goals, morality and competence in the prediction of recommendation for recruitment through perceived appropriateness of candidate. Supporting Hypothesis 1, we found that participants perceived a highly moral (as opposed to a low in morality) candidate as more appropriate for hire when the goals of the job were relational, while perceived appropriateness, in turn, predicted a stronger intention to recommend a candidate, supporting Hypothesis 2. Unexpectedly, the competence × type of goals interaction on perceived appropriateness of a candidate was not significant. A possible explanation for the insignificant result might be the manipulation of competence in the vignettes. Indeed, the hypothetical nature of the vignettes might have made it difficult to manipulate competence (high vs. low) sufficiently well to expect an interaction with type of goals (see also Randall and Gibson, 1990; Wason et al., 2002 about low realism of experimental vignettes). To achieve a more realistic set-up future research should consider laboratory experiments where a confederate takes the role of the candidate. However, it is worth mentioning that we found competence to interact with type of goals in the prediction of recommendation of a candidate for hire directly, suggesting that when an organization had instrumental goals competence predicted hiring recommendations more strongly than when an organization had relational goals. These results suggest that the person perception × type of goals interactive effect might directly influence recommendation for recruitment, and that this relationship does not necessarily emerge through the perceived appropriateness of candidate. Future research should further investigate these relationships.

Interestingly, the type of goals × morality × competence interaction, when predicting appropriateness of candidate, was significant showing that individuals deem a candidate who is high in competence and low in morality as more appropriate for hire when organizational goals are instrumental. In contrast, when organizational goals are relational, participants deemed a candidate who scored high in both dimensions (morality and competence) more appropriate. Importantly, perceived appropriateness fully mediated these effects on recommendation for recruitment. These results are further discussed in the General discussion.

## General discussion

Organizations are increasingly concerned with attracting and selecting the right types of employees (Combs et al., 2006). Employers place special attention on employee capabilities, skills, and knowledge while making recruitment decisions (Breaugh and Starke, 2000). Nevertheless, the literature points out the prevalence of people’s morality over competence when judging others and forming impressions about them (Abele, 2003; Abele and Wojciszke, 2007; Abele and Bruckmüller, 2011; Brambilla et al., 2012, 2013) because morality is more tightly connected with one’s character (Goodwin et al., 2014). In the current study we investigated the effects of a candidate’s competence vs. morality on the perceived appropriateness of a candidate for a job and in turn, on recommendation for recruitment. To do so, we took into consideration the moderating role of organizational goals (relational vs. instrumental; see Luce and Raiffa, 1957; Thibaut and Kelley, 1959; Olson, 1965; Tajfel and Turner, 1979; Turner et al., 1987; Deci and Ryan, 2000) and we hypothesized that candidates with superior morality over competence will be seen as more appropriate for hire and will be, in turn, recommended for hire when the goals of the hiring organization are relational. In contrast, we expected that a candidate of superior competence over morality would be deemed more appropriate and would be recommended for recruitment when the organization’s goals are instrumental.

Study 1 was a field study, aiming to test the effect of organizational values on the perception of a moral vs. competent candidate as appropriate for a job. Results provided support for Hypothesis 1 and showed that the more organizational goals are seen as relational the more a moral candidate is perceived to be appropriate for hire. Furthermore, the more an organization’s goals are instrumental, the more a competent candidate is perceived to be appropriate. Study 2 tested this hypothesis experimentally and showed that when a candidate is high in morality (although low in competence), a candidate is seen in a more positive light in terms of appropriateness for recruitment as compared to a candidate who is low in morality (but high in competence) under the condition that the hiring organization has relational goals. On the contrary, when an organization has instrumental goals, a candidate who is high in competence (although low in morality) is considered more appropriate for hire than a highly moral (but incompetent) candidate. Supporting Hypothesis 2, perceived appropriateness of the candidate, in turn, predicted intention for recommendation of the candidate.

These findings are in line with our postulation that morality is not always and unconditionally more important over competence and that the primacy effect of morality might get reversed in certain situations. Indeed, according to Wojciszke and Abele (2008), in certain contexts, such as at the work environment, competence may outweigh morality as it best serves the perceivers’ goals. Study 3 manipulated morality and competence separately as there is evidence that the two constructs are separate dimensions (Cislak and Wojciszke, 2008). Study 3 largely replicated the results of Study 2 and further found the type of goals × morality × competence interaction to be significant when predicting perceived appropriateness of candidate. Although we did not state specific hypotheses regarding this three-way interaction effect, the findings that occurred are noteworthy. More specifically, Study 3 showed that participants deem a candidate who is high in competence and low in morality more appropriate when organizational goals are instrumental. This finding is, to some extent, in line with the compensation effect (Wojciszke, 1994; Judd et al., 2005; Kervyn et al., 2012) which suggest a negative relationship between competence and morality and further extends it as it reveals the “utility” of scoring high in one dimension and low in the other. In other words, it is likely that a candidate who scores low in morality is seen as even more competent (according to the compensation effect) and hence, is perceived as more appropriate for a job when goals are highly instrumental. Moreover, Study 3 showed that individuals deem a candidate who scores high both in competence and morality to be more appropriate for hire when organizational goals are relational. To some extent, this finding is in line with the halo effect (Dion et al., 1972) which suggests a positive relationship between the two dimensions. A possible explanation of this result is that at work, even when relational goals are more dominant, candidate competence remains important due to the inherent instrumental nature of work (after all, one needs to be capable of performing according to given standards). Future research, including various methodological approaches is needed to further investigate the observed effects.

### Theoretical implications

These results have important theoretical implications. First, despite abundant research on the Big Two theory and the primacy effect of morality over competence (Peeters, 1983; Peeters and Czapinski, 1990; Peeters, 2001; Abele et al., 2021, see also Brambilla et al., 2012, 2013; Fousiani and van Prooijen, 2019; Unkelbach et al., 2020) there is very little empirical evidence on the importance of morality vs. competence in an organizational context. For instance, Pagliaro et al. (2013) showed that morality rather than competence-related information determined people’s behavioural inclination to be cooperative and help others in the workplace. Yet, to the best of our knowledge there is no research on the effects of morality vs. competence on people’s hiring decisions. The current study sheds light on this matter by investigating the moderating role of organizational goals. Indeed, depending on their culture, values, and identity (Hatch and Schultz, 1997) organizations may differ in terms of the goals that they promote (Lievens and Highhouse, 2003; Zhu et al., 2021). This study showed that the primacy of morality or competence in the hiring process depends on the goals of an organization. The current findings paint a clearer picture of the issue at hand, which despite its major role in organizational functioning, has been largely overlooked. Second, the current findings also speak to the Big Two theory (Abele and Bruckmüller, 2011; Abele and Wojciszke, 2013) and the person perception literature in general (Abele, 2003; Abele and Wojciszke, 2007; Abele and Bruckmüller, 2011; Abele et al., 2016) including the halo effect (Dion et al., 1972) and the compensation effect (Wojciszke, 1994; Judd et al., 2005; Kervyn et al., 2012; see also Moscatelli et al., 2019) and provide evidence about the conditions under which the primacy effect of morality can be reversed (see also, Wojciszke and Abele, 2008). Third, the current findings speak to the literature on relational (Brewer, 1979; Baumeister and Leary, 1995; Deci and Ryan, 2000) and rational decision-making (Luce and Raiffa, 1957; Thibaut and Kelley, 1959; Olson, 1965) and reveal the primacy effect of morality when decision-making is relationships-oriented and the primacy effect of competence when decision-making is rationality-based. Finally, this study provides evidence for the underlying mechanisms that drive the investigated effects. More specifically, in all the three studies we found evidence that the perceived appropriateness of a candidate (i.e., positive global impression of a candidate) was the mechanism that drove the moderated effect of morality vs. competence on recommendation intention. Accordingly, this contribution informs the HRM literature about the explanatory mechanisms that drive people’s hiring decisions in organizations.

### Practical implications

Apart from its theoretical implications, this study also features several – albeit tentative – practical implications: HR practitioners should be aware of people’s overall preference for socializing with moral as opposed to competent individuals as the former can be trusted to a greater extent and can contribute to the group harmony. Nevertheless, HR practitioners should not neglect the role that organizational goals play in the personnel selection process. Alternatively put, HR practitioners should be aware of their tendency to hire personnel that matches the organizational goals and be mindful of the consequences of such decisions. For instance, although organizational goals might be very important to consider when recruiting personnel, HR practitioners might make biased hiring decisions when overly influenced by the organization’s goals. Future research should further investigate the effects –both positive and negative-- of such hiring decisions on an organization’s functionality. Besides HR practitioners, these findings are important to employees in general as they reveal that the evaluation of employees’ core traits, namely morality and competence, is contingent on contextual characteristics and therefore, whether one or the other trait are evaluated in a positive or in a negative light depends on the context at hand.

### Limitations and future directions

Although we used different methods to operationalize our variables across the studies (Study 1: field study, Study2: 2 × 2 experiment, and Study 3: 2 × 2 × 2 experiment) the findings were largely similar revealing the robustness of the investigated effects. Yet, this work includes a number of limitations and inconsistencies between the three studies. A significant limitation of Study 1 (field study) is its cross-sectional design (without time-lag between the several measures) and therefore did not test the mediating effect that was hypothesized in Hypothesis 2 (see Podsakoff et al., 2012 for the risks of common-method bias in cross-sectional research). Studies 2 and 3 illuminate the mediation effect, yet these studies are experiments relying on vignettes that are hypothetical in nature. Although our vignettes were adapted versions of vignettes that have been successfully used in previous research (Laustsen and Bor, 2017), conclusions drawn from Studies 2 and 3 are only about perceptions and may not transfer to real-life situations. Moreover, although participants of Study 1 were recruiters having HR experience, in Studies 2 and 3 we used convenient samples that might lack such professional experience. Accordingly, we cannot generalize with certainty the findings of Studies 2 and 3 to the broader HR and recruiter community. The current research takes first steps in challenging the notion that morality has a primacy effect over competence and addresses the question whether organizational goals moderate the effect of competence and morality of a candidate on perceived appropriateness and intention to hire a candidate. This contribution hence needs to be seen as a preliminary step towards a more fine-grained understanding of the relationship between morality and competence perceptions on the one hand and people’s judgement on the other hand. Future research needs to include a broader range of methodological designs (e.g., time-lagged field studies) in order to shed light on this topic.

Moreover, one inconsistency that we observed is that, the main effect of morality and competence on perceived appropriateness of the candidate was not significant in Study 2 but it proved to be significant in Study 3. This might be due to the different experimental design that we used in the two studies; Study 2 directly compared competence vs. morality of a candidate whereas Study 3 manipulated the two variables separately. Moreover, the competence × type of goals interaction effect on perceived appropriateness of a candidate was not significant in Study 3, while this interaction was significant when recommendation of a candidate was the outcome variable.

Finally, all our measures are self-reported, non-behavioral measures and therefore, we cannot conclude with certainty whether the observed effects can be generalized to people’s behavior. Future research should further investigate these effects with alternative tools and behavioral measures (e.g., actual recruitment of job candidates) for a better understanding of the effects of morality and competence on work-related decisions.

### Concluding remarks

A growing body of research has underscored the primacy effect of morality over competence in person perception. Morality informs people about whether or not a target is a threat and it is more diagnostic of behavioral intentions (Leach et al., 2007; Brambilla et al., 2012, 2013; see also Fousiani and van Prooijen, 2019). While we do not dispute that this moral primacy effect is likely to occur in most situations, it is important to be aware of the boundary conditions of this effect. The present research sought to clarify that the primacy effect of morality (or competence) in social judgment largely depends on an observer’s goals. Apparently, when organizations have goals that require high competence among employees (e.g., profit maximization), people may prioritize a candidate’s competence over morality in the recruitment process.

## Data availability statement

The datasets presented in this study can be found in online repositories. The names of the repository/repositories and accession number(s) can be found at: https://osf.io/nduxs/?view_only=22a7b18f2ccc4df1b0f051665fd9fb72(OSF).

## Ethics statement

The studies involving human participants were reviewed and approved by Ethical Committee of the University of Groningen, Nr PSY-2021-S-0507. The patients/participants provided their written informed consent to participate in this study.

## Author contributions

KF: writing first draft of manuscript, research design, data collection, and statistical analyses. J-WP: research design and edits in the manuscript. BA: research design, edits in the manuscript, and data collection. All authors contributed to the article and approved the submitted version.

## Conflict of interest

The authors declare that the research was conducted in the absence of any commercial or financial relationships that could be construed as a potential conflict of interest.

## Publisher’s note

All claims expressed in this article are solely those of the authors and do not necessarily represent those of their affiliated organizations, or those of the publisher, the editors and the reviewers. Any product that may be evaluated in this article, or claim that may be made by its manufacturer, is not guaranteed or endorsed by the publisher.

## Supplementary material

The Supplementary material for this article can be found online at: https://www.frontiersin.org/articles/10.3389/fpsyg.2022.923329/full#supplementary-material

## Footnotes

^1^The Big Two Theory identifies an additional trait, namely warmth or sociability (e.g., friendliness), which along with morality belongs to the broader category of communion (Abele and Wojciszke, 2007). Yet, the morality facet of communion has a more important role in person perception than the warmth/sociability facet (Leach et al., 2007; Brambilla et al., 2011, 2012, 2013; Brambilla and Leach, 2014; Kervyn et al., 2015), and therefore in the current work we only focused on morality.

^2^Besides perceived appropriateness of candidate, in this study we also measured recommendation for recruitment of candidate. However, given the cross-sectional nature of Study 1 we did not test the mediating role of perceived appropriateness in the relationship between candidate traits (competence and morality) by organizational goals and recommendation for recruitment (Hypothesis 2). Yet, we report the effects on recommendation for recruitment in the Additional Exploratory Analyses in the Online Supplementary material. These analyses further support our line of reasoning.

^3^Results in all the three studies are similar when control variables are not included in the analysis.

^4^A total of 586 participants clicked on the Qualtrics link but only 318 completed it. Therefore, 268 participants who did not provide any answers to the survey questions were excluded from the dataset.

^5^We should report that employment status of participants did not influence either appropriateness of the candidate or recommendation for recruitment.

^6^The experimental design and the hypothesized interaction effect (Hypothesis 1) were preregistered; However, the mediation hypothesis (Hypothesis 2) was not yet preregistered for Study 2 (only for Study 3). Moreover, in the preregistration we included an additional outcome variable (e.g., rejection of a candidate). The effects on that outcome variable did not come out significant and therefore, for the sake of brevity, we decided to exclude it from the paper (and also did not include the measure in Study 3).

^7^In order to make our manipulations more convincing, participants were asked to rate the appropriateness of both applicants and indicate their intention to recommend them for recruitment. However, in the current paper we only focus on ratings of the candidate whose morality and competence varied.

^8^Besides the ipsative scales that we used for the assessment of perceived appropriateness and recommendation of a candidate, we included Likert scales assessing the perceived appropriateness and recommendation of moral and competent candidates. Unsurprisingly, we observed a high ceiling effect and we decided to discard these scales from the study.

^9^ https://osf.io/293py/?view_only=976bdcd813ea426d8ab469b0066dce6a
