# Supplementary material for: Appearing competent or moral? The role of organizational goals in the evaluation of candidates
Source: Front Psychol. 2022 Sep 12;13:923329. doi: 10.3389/fpsyg.2022.923329 (PMC9513611; doi:10.3389/fpsyg.2022.923329)
Supplement: Supplementary file 1 [file Data_Sheet_1.docx]

**Study 1**

**Relational and Instrumental Goals**

**Relational Goals/ Concerns** (inspired by the face concerns scale of Ting-Toomey & Oetzel (2001) (1 = strongly disagree to 7 = strongly agree)

Every department or company have certain values and goals that they emphasize and prioritize over others. Please rate each of the following values based on their importance to your department/company.

The department/company where I work…

1. …is primarily concerned with protecting the feelings of the people who work there.
2. …emphasizes relationship harmony.
3. …is concerned with maintaining peace in the interaction between people (employees, clients etc.)
4. …values a peaceful resolution to conflict between people.
5. …is concerned with preserving staff members’ harmonious communication.
6. …wants to make people who work there feel good.
7. …wants to establish a positive relationship with the people who work there.
8. …is concerned with developing processes, routines, and tools to optimize relationship quality between the members of the department/company.
9. …invests in people’s well-being and sense of belonging.
10. …wants to have long-term relationships with people who work there.

**Instrumental Goals/Concerns** (inspired by the Instrumental concern scale of Wilson & Putnam, (1990) (1 = strongly disagree to 7 = strongly agree)

1. …is primarily concerned with reaching outcomes that would benefit the company/department.
2. …is mostly concerned with achieving high profits.
3. …is concerned with maximizing its revenues and total benefit.
4. …focuses on finding solutions that would maximize its profits.
5. …values success and profit.
6. …is concerned with how to make “good” deals.
7. …is concerned with developing processes, routines, and tools to optimize profit.
8. …is concerned with finding profitable solutions to financial issues.

**Appropriateness of Candidate (Based on the Agency and Communion Scale of Abele & Wojciszke, (2007).**

While answering the following questions, please bring to mind situations of job candidates who do meet the job requirements. In this study, we are not interested in how your company/department would evaluate candidates who do not meet the job requirements.

Which candidates do you see as more appropriate for hire in your company/department?

In the company/department where I work, I would see as more appropriate (fitting) a candidate who is:

1= moral, 7= competent

1= sincere, 7= skillful

1= righteous, 7 = intelligent

1= honest, 7 = capable

1= fair, 7 = efficient

**Intention to Hire or Recommend for Hire**

Which candidate would you hire or recommend for recruitment in your company/department?

**I would hire or recommend for recruitment in the company/department where I work, a candidate who is:**

1= moral, 7= competent

1= sincere, 7= skillful

1= righteous, 7 = intelligent

1= honest, 7 = capable

1= fair, 7 = efficient

**Studies 2 and 3**

Imagine that you are the CEO of a big software company. The company has recently posted a job vacancy to recruit a Management consultant. The company has very specific requirements regarding the person to be recruited. Please read the job description carefully to see the requirements for the successful candidate.

**Manipulation of Goals - Vignettes**

**Instrumental Goals:**

**_____________________________________________________________________**

IntraCOM is a large and rapidly growing software company in downtown London that offers an award-winning product suite that is transforming how organizations work. You are seeking a candidate to fulfill the Management Consultant role in the company. The successful candidate will be responsible for a range of financial profit-related activities. This position requires the following abilities:

 -Designing a strategic plan for the maximization of the company’s revenues and financial profit.

-Identifying financial problems and designing economically profitable strategies.

-Establishing and achieving financial profit-related objectives.

-Developing processes, routines, and tools to optimize the financial profit of the company.

  The motto of the company is: “Financial profit and success always come first!”

The successful candidate is expected to actively promote the company’s interests by identifying and making visible financial problems and suggest effective financial solutions.

 Application deadline: 25 February 2021

 _________________________________________________________________________

**Relational Goals:**

**________________________________________________________________________**

IntraCOM is a large and rapidly growing software company in downtown London that offers an award-winning product suite that is transforming how organizations work. You are seeking a candidate to fulfill the Management Consultant role in the company. The successful candidate will be responsible for a range of relationship-related activities. This position requires the following abilities:

 -Designing a strategic plan for the maximization of the employees’ well-being and sense of belonging to the company.
-Identifying collaboration problems between company members and designing conflict resolution strategies.
-Establishing and achieving relationship-related objectives.
-Developing processes, routines, and tools to optimize relationship quality between the members of the company.

 The motto of the company is: “Connecting people and building relationships always come first!”

The successful candidate is expected to actively promote the company’s interests by identifying and making visible collaboration/relation problems and suggest effective communication solutions.

Application deadline: 25 February 2021

 _**__________________________________________________________________________**

**Manipulation Checks for Goals**

**Based on what you have just read, the job vacancy has a focus on:**

A sense of belonging, fair solutions, communication

Revenues and financial profit maximization

(1= not at all true, 7 = absolutely true)

**Manipulation of Morality/Competence**

**Superior Competence and Inferior Morality:**

**Mr de Vries** has many years of experience in management. He has designed an excellent initiative to identify and analyze company’s interests and translate them into projects that assure their realization. His ideas have been widely praised for their insights, effectiveness, and originality.

However, in his career so far, Mr de Vries has proved to be a controversial person. His colleagues describe him as not the most honest man who sometimes breaks his word. Mr de Vries is also accused of being, at times, disrespectful or impolite.

**Superior Morality and Inferior Competence:**

**Mr**de Vries has mostly worked as a security engineer and has very little experience in management. He frequently recites an initiative to identify and analyze company’s interests and translate them into projects that assure their realization. However, his ideas have been widely judged as shallow, ineffective, and naïve.

 However, in his career so far, Mr de Vries has proved to be a reputable person. His colleagues describe him as an honest man who always keeps his word. Mr de Vries is also praised for treating others with respect and politeness.

**(Moderate) Competence/Morality of the Other Candidate**

The other candidate,**Mr Van Someren** has been active in the role of manager for the last few years. He has demonstrated considerable expertise in business in general and in management in particular, but some of his colleagues mention that his experience and skills in other, perhaps more pressing issues are less obvious.

 Moreover, his colleagues consider Mr Van Someren to be a reliable person who is easy to work with. That said, some mention that he had to leave his previous job due to personal conflicts with his former employer.

**Manipulation Checks for Competence/ Morality**

**Based on what you have just read**, to what extent do you perceive **Mr de Vries** as:

**Competent, intelligent, skilled, moral, sincere, honest (1= not at all, 7 = a lot)**

**Measures**

**Recommendation of the Candidate for the Job** (based on Higgins and Judge, 2004).:

Would you recommend Mr de Vries for this position?

Would you offer this job to Mr de Vries?

Would you recommend this applicant for recruitment? (this item added in Study 3 only)

(1 = absolutely not, 7 = absolutely yes)

**Global Impression about the Candidate (Perceived Appropriateness of the Candidate; based on Cable and Judge (1997) and Brambilla et al. (2012):**

To what extent do you think that Mr de Vries fulfills the criteria for this job?

To what extent do you think that Mr de Vries is an appropriate candidate for this position?

Do you think the applicant is a good fit for the job? (this item was added in Study 3 only)

(1= not at all, 7 = to a great extent)

**Additional Exploratory Analyses**

**Study 1**

**Recommend for Recruitment (intention to hire).** Similar to the appropriateness of the candidate scale, we developed a 5-item bipolar scale assessing what traits of job candidates would make respondents hire or recommend them for recruitment to the department or company where they work. One pole was presenting morality-related traits (1= *moral, sincere, righteous, honest, fair*) and the other pole was presenting competence-related traits (7= *competent, skillful, intelligent, capable, efficient*).

**Results**

We tested the effect of organizational goals on respondents’ intention for recommending or hiring a moral or competent candidate via regression analysis. As predicted, the effect of relational goals on the intention to recommend or hire a moral or competent candidate came out significant showing that participants recommended for hire a moral candidate to the extent that the organization’s goals were more strongly relational. The effect of instrumental goals on the intention to recommend or hire a moral or competent candidate was also significant showing that when the organization goals are instrumental, participants have a stronger intention to recommend them for recruitment or hire them (see Table 6).

**Table 6**

*Regression Analyses Results on Perceived Appropriateness of a Moral or Competent Candidate and Recommendation for Recruitment (Study 1).*

| Predictor | *B* | *SE* | *t* | *p* | 95% *CI* |
| --- | --- | --- | --- | --- | --- |
| Recommendation Intention^b^ | | | | | |
| Constant | 5.53 | 0.38 | 14.53 | <.001 | 4.78; 6.30 |
| Relational Goals^a^ | -0.18 | 0.06 | -3.04 | .003 | -0.30; -0.07 |
| Instrumental Goals^a^ | 0.13 | 0.05 | 2.83 | .005 | 0.04; 0.22 |

^a^Rrelational and instrumental goals were rated on a 7-point (1 = *strongly disagree* to 7 = *strongly agree*) Likert scale. ^bI^ntention for recommendation for recruitment were rated on a 7-point (1= *moral candidate*, 7= *competent candidate*) bipolar scale.
