# Supplementary material for: Appearing competent or moral? The role of organizational goals in the evaluation of candidates
Source: Front Psychol. 2022 Sep 12;13:923329. doi: 10.3389/fpsyg.2022.923329 (PMC9513611; doi:10.3389/fpsyg.2022.923329)
Supplement: Supplementary file 2 [file Table_1.docx]

**Appendix A**

*Results From a Factor Analysis of the Relational and Instrumental Goals Scales (Study 1).*

| Item | Factor 1 | Factor 2 |
| --- | --- | --- |
| Factor 1: Relational Goals |  |  |
| The department/company where I work… |  |  |
| …is primarily concerned with protecting the feelings of the people who work there. | **.69** | .04 |
| …emphasizes relationship harmony. | **.83** | -.03 |
| …is concerned with maintaining peace in the interaction between people (employees, clients etc.) | **.73** | -.004 |
| …values a peaceful resolution to conflict between people. | **.78** | -.02 |
| …is concerned with preserving staff members’ harmonious communication. | **.86** | .003 |
| …wants to make people who work there feel good. | **.83** | -.03 |
| …wants to establish a positive relationship with the people who work there. | **.87** | -.08 |
| …is concerned with developing processes, routines, and tools to optimize relationship quality between the members of the department/company. | **.72** | -.02 |
| …invests in people’s well-being and sense of belonging. | **.84** | -.05 |
| …wants to have long-term relationships with people who work there. | **.80** | -.10 |
| Factor 2: Instrumental Goals |  |  |
| …is primarily concerned with reaching outcomes that would benefit the company/department. | -.06 | **.89** |
| …is mostly concerned with achieving high profits. | -.16 | **.90** |
| …is concerned with maximizing its revenues and total benefit. | .022 | **.90** |
| …focuses on finding solutions that would maximize its profits. | .14 | **.88** |
| …prioritizes success and goal achievement. | .52 | .32 |
| …values success and profit. | -.06 | **.89** |
| …is concerned with how to make “good” deals. | -.01 | **.89** |
| …is concerned with developing processes, routines, and tools to optimize profit. | .18 | **.79** |
| …is concerned with finding profitable solutions to financial issues. | .10 | **.87** |
| …usually conceals the truth about problems of weaknesses it has. | -.27 | .32 |
| …withholds information that could put it to a disadvantage. | -.30 | .44 |

*Note.* The extraction method was principal axis factoring with rotation. Factor loadings above .50 are in bold. Items with loadings below .50 were excluded from the final scale. We separately ran the analyses including all the items and the results were the same as the currently reported.
